# Relapsed angioimmunoblastic T-cell lymphoma with acquired expression of CD20: a case report and review of the literature

**DOI:** 10.1186/1472-6890-13-18

**Published:** 2013-06-05

**Authors:** Yara Banz, Fatime Krasniqi, Stephan Dirnhofer, Alexander Tzankov

**Affiliations:** 1 Institute of Pathology, University of Bern, Bern, Switzerland; 2 Department of Oncology, University Hospital Basel, Basel, Switzerland; 3 Institute of Pathology, University Hospital Basel, Schönbeinstrasse 4, 4031, Basel, Switzerland

**Keywords:** Angioimmunoblastic T-cell lymphoma, Lineage infidelity, CD20, Tumour-cell rich

## Abstract

**Background:**

Angioimmunoblastic T-cell lymphoma is one of the most common types of peripheral T-cell lymphomas, usually presenting at an older age with an aggressive clinical course. Its characteristic morphological presentation and follicular helper T-cell phenotype help to distinguish it from other T-cell lymphomas.

**Case presentation:**

We recently encountered the unique case of a 63-year old patient with relapsed tumour-cell rich angioimmunoblastic T-cell lymphoma, presenting with a “classical” phenotype and, in addition, an acquired, strong, aberrant expression of CD20.

“Lineage infidelity” of phenotypic markers is a well-documented phenomenon in lymphomas and leukemias, a circumstance currently still poorly understood and with the potential to bring about erroneous interpretations, causing diagnostic havoc. This case represents one of the few documented angioimmunoblastic T-cell lymphomas with strong CD20 expression. Of interest, CD20 expression was only detected in the recurrent lymphoma and not upon initial diagnosis. The clinical importance of this finding lies in the potential for treatment with an anti-CD20 antibody, for instance Rituximab, in addition to standard chemotherapy protocols for angioimmunoblastic T-cell lymphoma.

**Conclusion:**

Diagnostic work-up of lymphomas to determine their lineage should therefore consider morphology, pheno- as well as genotypic characteristics, where appropriate, and in particular signs of progression and change in marker profile in relapsed cases e.g. acquisition of “non-lineage” markers such as CD20 in T-cell lymphoma.

## Background

Angioimmunoblastic T-cell lymphoma (AITL) represents one of the most common specific subtypes of peripheral T-cell lymphomas, making up roughly 20% of all cases [1]. The disease usually follows an aggressive course, with poor overall survival of the mainly elderly and frequently male patients [2]. The presentation and immunophenotype is often very characteristic, typified by effacement of the normal lymph node architecture by a proliferation of small to medium-sized lymphocytes with pale cytoplasm and minimal atypia intermingled with a more or less pronounced non-neoplastic, reactive, background infiltration of T-cells, B-cells, and plasma cells, and often sparing the peripheral cortical sinuses. Furthermore, an abundance of high endothelial vascular structures as well as expanded follicular dendritic cell meshworks are observed. The neoplastic cells present with a follicular helper T-cell (T_FH_) phenotype with positivity for CD3, CD4, CD10, PD-1 and CXCL13 [3]. The derivation of AITL from T_FH_ may also explain the commonly observed expansion of B-cells in the setting of angioimmunoblastic T-cell lymphoma, since these T_FH_ play a key regulatory role in the germinal center processing of B cells [4].

Aberrant expression of so-called “lineage specific” markers in lymphomas and leukaemias is a well-known phenomenon and expression of CD20 in T-cell lymphomas is well documented [5]. However, we herein report one of only very few published case observations of CD20 expression in AITL and the second case ever of acquired CD20 expression in relapsed AITL. An aberrant and unexpected immunophenotype may lead to substantial diagnostic difficulties. However this is also of clinical importance, offering for instance the possibility of an anti-CD20 therapy such as Rituximab in addition to standard treatment protocols.

In the following, the authors discuss the case of relapsed AITL presenting with an unusual tumour-cell rich phenotype and aberrant CD20 expression upon relapse.

## Case presentation

A 63-year old male patient presented with generalized lymphadenopathy, exanthema, anasarca and overall weakness in November 2011. His personal history was remarkable for the diagnosis of a peripheral T-cell lymphoma, not otherwise specified (NOS) seven years prior (initial Ann Arbor stage IIA) and subsequent treatment with eight cycles of chemotherapy (cyclophosphamide, hydroxydaunorubicin, oncovin and prednisone) followed by 30 Gy involved-field irradiation of the inguinal region. He had been in complete remission since completion of therapy. Currently, a diagnostic procedure was performed, including an excisional lymph node biopsy from the inguinal region.

### Materials and methods

The reported data are represented in an irreversibly anonymized manner and were obtained with the standard diagnostic processes without additional tissue exhaustion, according to the cantonal regulations. All research carried out on the human tissue samples were in compliance with the Helsinki Declaration. The excised lymph node tissue was immediately fixed in 4% buffered formalin. Following complete fixation, the sample was paraffin-embedded and routinely stained with haematoxylin-eosin, Giemsa, Periodic acid-Schiff’s reaction and Novotny.

### Immunohistochemistry

Immunohistochemistry was performed on formalin-fixed, paraffin-embedded serial tissue sections. For antigen retrieval, tissue sections were immersed and microwaved in citrate buffer (pH 6.0) as appropriate for each antibody. After rinsing with phosphate-buffered saline, immunohistochemical analysis was performed using antibodies against CD2, CD3, CD4, CD5, CD7, CD8, CD30, CD19, CD20, CD79a, PAX-5, ALK1, PD-1 and CXCL13 (all antibodies were from Ventana except CD19 (Dako) and CXCL13 (R&D) and all were pre-diluted/ready to use, except for CXCL13, which was diluted 1:50); respective clones: MRQ11, 2GV6, SP35, SP19, SP94, SP57, L29, LECD19, BerH2, SP18, SP34, ALK01, MRQ22 and polyclonal AF801). All staining was performed on an automated immunostainer (Benchmark XT from Ventana/Roche, USA) using a streptavidin-biotin peroxidase detection system, except for CXCL13, which was incubated manually for 2 h at 37°C and also detected using a streptavidin-biotin peroxidase detection.

For detection of Epstein-Barr virus-encoded RNA (EBER) an in situ hybridization was performed on formalin-fixed, paraffin-embedded tissue sections using the Ventana ready-to-use kit as prescribed by the manufacturer.

### Molecular pathology

To assess T- and B-cell clonality and clonal relationships between the T-cell lymphomas diagnosed in 2004 and 2011 polymerase chain reaction (PCR) for both *T-cell receptor gamma* and IgH heavy chain (*IGH*) was performed on formalin-fixed, paraffin-embedded whole tissue sections.

Genomic DNA was extracted using a Genovison extraction kit (Qiagen, Germany). Polymerase chain reaction (PCR) based analysis for *IGH* gene rearrangements was performed utilizing consensus FR1, FR3 and J primers, as previously described [6]. The PCR products were examined using a high-resolution fragment length analyzer (ABI 310 Genetic Analyzer, Applied Biosystems/Life Technologies, USA). Monoclonal gene rearrangements were identified as prominent, single-sized amplification products; the base pair length was recorded for each fraction. A shift of the PCR products of more than three base pairs between the cases was considered to indicate a clonally unrelated event.

### Histological findings

**Figure 1:**
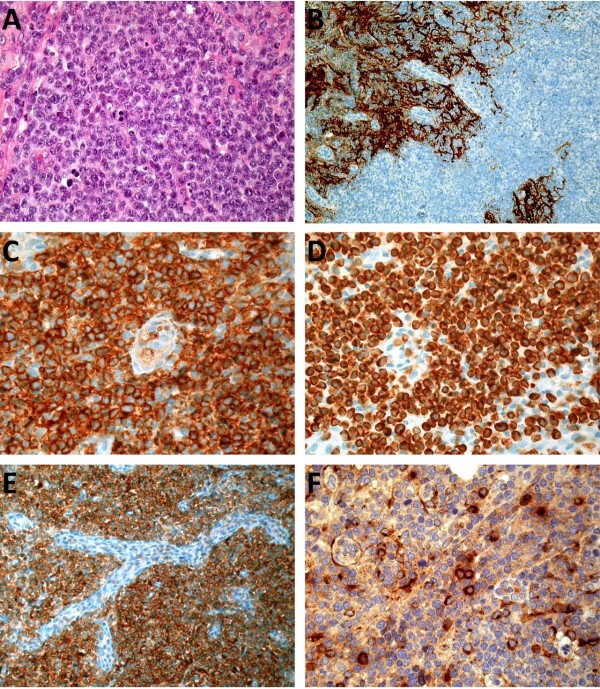
**Hematoxylin and eosin (A) as well as immunochemical stainings (B-F) of the current lymph node biopsy from 2011.** Effacement of the normal lymph node architecture by medium-sized atypical lymphocytes. Evidence of expanded mesh works of follicular dendritic cells stained by CD23 (**B**). Neoplastic cells show strong positivity for CD3 (**C**) and CD4 (**D**) as well as positivity for PD-1 (**E**) and CXCL13 (**F**).

Regular histology revealed effacement of the normal lymph node architecture by a vaguely nodular to diffuse, tumour-cell rich lymphoid infiltrate with focal sparing of peripheral cortical sinuses and destruction of the lymph node capsule. An abundance of high endothelial venules was noted (Figure 1A). The neoplastic cells consisted of medium sized atypical lymphocytes with slightly eccentrically located nuclei with coarse chromatin. The mitotic count was elevated (>30/10 high power fields, HPF).

### Immunohistochemical studies and in situ hybridization of the current biopsy

Immunochemistry revealed the neoplastic cells to be of a T-cell origin with positivity for CD2, CD3, CD4 and CD5, expression of PD1 (moderate staining intensity) and focal positivity for CXCL13 (Figure 1B-H); there was antigenic loss for CD7. Furthermore the cells strongly and diffusely expressed CD20, but no other B-cell markers (CD79a, CD19 and PAX5), which stained intermingled reactive small B-lymphocytes and scattered immunoblasts. CD8 highlighted isolated non-neoplastic T-lymphocytes. ALK1 and CD30 were negative. CD23 exposed expanded follicular dendritic cell mesh works. EBER in situ hybridization did not reveal EBV infected tumour cells and only isolated infected B-cells.

### Molecular pathology

**Figure 2:**
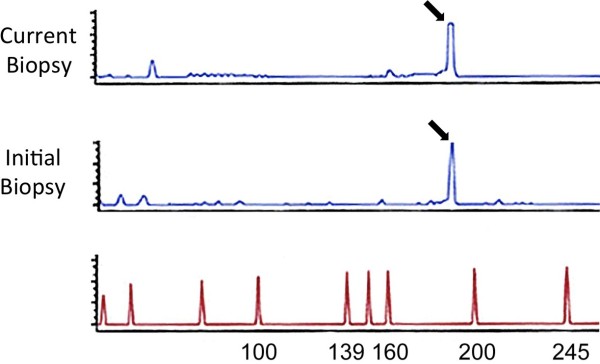
**Examination of the polymerase chain reaction (PCR) for T-cell receptor gamma from DNA extracted from formalin-fixed, paraffin-embedded whole tissue sections (current lymph node as well as tissue from the initial diagnosis) using a high-resolution fragment length analyser.** Monoclonal gene rearrangements are identified as prominent, single-sized amplification products. This is seen in both samples, with a fragment length analysis showing a “peak” (black arrow) at 191 base pairs length in two subsequent runs, suggesting clonally-related relapse. The bottom line (red) reveals the size of the fragment size.

Molecular pathology performed on the current lymph node sample revealed a monoclonal T-cell population based on *T-cell receptor gamma* fragment length analysis, showing 191 base pairs length in two subsequent runs. Retrospectively the same population was detected in the initial lymph node biopsy obtained seven years previously, suggesting a clonally related relapse (Figure 2). Cytogenetic analysis was not performed. B-cell clonality analysis was performed in the initial biopsy as well as in the follow-up biopsy after detection of CD20 expression in the neoplastic population to exclude progression to or concomitant existence of B-cell lymphoma. Clonal B-cells were not detectable in either of the tested samples. At this time point the diagnosis of relapsing AITL was made. Despite the clear-cut positivity of the tumour cells for the B-cell marker CD20, progression to frank B-cell lymphoma, which can be occasionally observed in AITL, could be excluded taking into consideration histopathology and phenotyping as well as results of the B-cell clonality testing and in particular results from the T-cell clonality analysis, which revealed an identical clone in the initial biopsy as well as in the tumour relapse.

### Retrospective immunohistochemical studies of the initial biopsy

**Figure 3:**
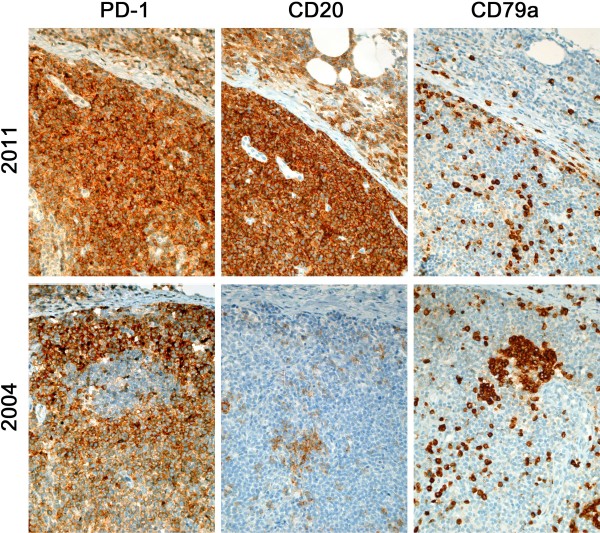
**Immunochemical stainings of the lymph node biopsy form 2011 (top row) as well as from the initial biopsy from 2004 (bottom row).** In the top row, PD-1 and CD20 equally mark the neoplastic T-cells, whilst staining for CD79a provides the contrast to CD20, highlighting the sparse B-cell infiltrate. Only CD20-CD79a double positive cells represent B-cells, the (PD-1 positive) CD20 positive, CD79a negative cells effectively represent the neoplastic T-cell population. In the initial lymph node biopsy in the bottom row retrospective PD-1 staining marks the neoplastic T-cells. In contrast to the differing staining pattern in the top row, the CD20 and CD79a mark a different population of cells, namely an infiltrate of reactive B-cells.

The initial lymph node biopsy was retrospectively analysed for the expression of CD20 in light of the suspected lymphoma relapse. The initial biopsy showed features consistent with AITL (expression of CD4, sparing of peripheral cortical sinuses, slightly expanded follicular dendritic cell meshworks, abundance of high endothelial vascular structures), the blasts were CD10-negative (PD1 and CXCL13 were not yet available at the time of diagnosis in our laboratory), so that the diagnosis of a peripheral T-cell lymphoma, NOS was preferred at that time, which was also in line with the lack of the typical biological AITL syndrome, except for minimal exanthema. Retrospective analysis showed presence of PD1- and partially CXCL13-positive atypical cells in the initial biopsy. DNA extraction and PCR-based *T-cell receptor gamma* fragment length analysis revealed, as already mentioned, the same clonal T-cell population in the initial biopsy as in the relapse. Therefore the initial diagnosis was modified accordingly. Careful comparison between the B-cell markers (CD20, CD79a and PAX5) as well as the T-cell marker CD3 and marker for T_FH_ cells (PD-1) did not suggest co-expression of CD20 on the neoplastic T-cells at the time of initial diagnosis (Figure 3). Thus, we assume that expression of CD20 was acquired by the AITL at the time of relapse. In addition, morphological comparison between the initial biopsy from 2004 and the biopsy from 2011 displayed a significant increase in neoplastic cells in the latter compared to the former, but no cytomorphological signs of progression such as the presence of frank anaplasia of the tumour cells.

## Conclusions

We report the case of relapsed AITL, which, in addition to the “classical” follicular helper T-cell (T_FH_) phenotype [3], displayed a striking expression of CD20. Whilst expression of CD20 has been reported in T-cell lymphomas [6], this case is one of only very few published observations of CD20 expression in AITL and is unique in that the CD20 expression was acquired upon relapse. The first case of CD20-positive AITL was reported by Yokose et al. as peripheral T-cell lymphoma with clinical characteristics resembling angioimmunoblastic lymphadenopathy [7]. The second report was by Tachibana et al. in 2011 [8] with features very similar to the herein presented case, in particular also the acquisition of CD20 expression upon disease progression. Only recently another case was observed by Foukas et al. [9].

Remarkable in our case is the clear-cut positivity of the neoplastic T-cells for CD20, a B-cell marker, and the progression from classical AITL in 2004 to tumour-cell rich AITL in 2011. “Lineage infidelity” of phenotypic markers is a well-documented phenomenon in lymphomas [10-13]. Although the biological mechanisms and significance of this characteristic are poorly understood, it has the potential to cause diagnostic havoc. Therefore a multimodal approach that considers morphology, pheno- and genotypic characteristics is essential to achieve a final correct diagnosis. Hypothetically, CD20 positivity in T-cell lymphomas may include derivation from subsets of CD20 positive T-cells undergoing neoplastic transformation or CD20-acquisition following neoplastic transformation of the T-cells; the latter applying to our case, where CD20 was acquired upon relapse. Indeed, CD20 expression may be acquired in T-cell lymphomas following activation of the T-cells, as has been demonstrated in stimulated lymph nodes from monkeys with simian immunodeficiency virus [14]. This experimental evidence is supported by the current case as well as the series reported by Tachibana, Rahemtullah and colleagues [5,8] where the proportion of CD20/CD30 co-expressing T-cells increased over time. CD20 may therefore represent an “activation marker” acquired after neoplastic T-cell transformation. Another possible explanation for the observed CD20 acquisition may be trogocytosis [15]. Upon interaction with surrounding cells, lymphoid cells, especially CD8 positive cytotoxic lymphocytes and NK-cells, can initiate membrane bridges with target cells, thereby capturing small membrane patches from their interaction partners and in the process potentially acquiring “lineage-foreign” antigens [16]. Since at least in our case there were no morphological hallmarks of cell cannibalism or signs of hemophagocytosis, the AITL was of CD4 lineage and CD20 was the single “lineage-improper” antigen expressed by the malignant cells, we speculate that trogocytosis might not explain our observations.

Of clinical relevance is the possibility of targeting such cases with Rituximab, a chimeric murine/human monoclonal antibody directed against the CD20 antigen. Currently, our patient was treated (off-study) with high dose chemotherapy combining cisplatin with cytosine arabinoside and dexamethasone with addition of Rituximab (R-DHAP). This treatment regimen lead to a partial remission and the patient subsequently underwent autologous stem cell transplantation. However, the disease relapsed only two months later and he succumbed to sepsis in neutropenia upon salvage immunotherapy with Lenalidomide in preparation for allogeneic stem cell transplantation.

Treatment strategies incorporating the anti-CD20 antibody Rituximab have significantly improved results in patients with mature B-cell lymphomas [17]. And whilst Rituximab has been shown to suppress EBV-positive B-immunoblasts in the microenvironment of AITL [18], a recent small clinical trial revealed no clear survival benefit of adding Rituximab to conventional CHOP chemotherapy to target the intratumoral B-cells [19].

Potential diagnostic difficulties and pitfalls in this case were not only apparent because of the aberrant CD20 expression but also because of the unusual density of the tumour cell infiltrate, resulting in morphological overlap with peripheral T-cell lymphoma not otherwise specified (PTCL, NOS). An important clue to the correct diagnosis of AITL – also in the initial biopsy – was the expanded mesh works of follicular dendritic cells, highlighted by the CD23 stain and the characteristic phenotype with expression of T_FH_ markers. The differentiation between relapsed AITL with aberrant CD20 expression and AITL relapse as frank B-cell lymphoma, a particular possibility in such instances, can be accomplished by testing for and comparing T- and B-cell clonality both in the initial sample and upon relapse as well as by evaluation of the immunohistochemical profile of the neoplastic cells in serial sections.

Taken together this case highlights the spectrum by which AITL can present. From the “classical” form readily suspected in haematoxylin and eosin stains with a dispersed tumour-cell infiltrate sparing peripheral cortical sinuses, with expanded follicular dendritic cell mesh works and abundance of high endothelial vascular structures to the more tricky form mimicking PTCL, NOS with dense tumour-cell infiltrates, aberrant marker expression and low numbers of EBV-positive B-cells. Correct classification of peripheral T-cell lymphomas may become increasingly important and in particular the diagnosis of PTCL, NOS should not be used as a “waste basket” for tricky cases. Increasingly molecular profiling provides evidence that different pathways are primarily involved in different subsets of T-cell lymphomas. Indeed, as is the case with AITL, in which activation of the NF-κB pathway appears to be of importance [20], such new insights may even lead to more tailored treatment options in the future, including the use of inhibitors of the NF-κB pathway in AITL.

## Consent

Written, informed consent was obtained from the deceased’s next of kin for scientific post-mortem clinico-pathological work-up and publication of the anonymized data.

## Competing interests

The authors declare that they have no competing interests.

## Authors’ contributions

All authors were involved in conception, design and acquisition of the data, analysis and interpretation of the data and were directly involved in drafting as well as revising the manuscript. All authors read and approved the final manuscript.

## Pre-publication history

The pre-publication history for this paper can be accessed here:

http://www.biomedcentral.com/1472-6890/13/18/prepub
